# An Unusual Complication of Internal Hernia Post-laparoscopic Sleeve Gastrectomy: A Video Case Report

**DOI:** 10.7759/cureus.71146

**Published:** 2024-10-09

**Authors:** Talat Albeiti, Alwahhaj Khogeer, Aly Elbahrawy

**Affiliations:** 1 General Surgery, King Abdullah Medical City, Makkah, SAU; 2 Specialized Surgery, King Abdullah Medical City, Makkah, SAU

**Keywords:** bariatric surgery complications, gastric bypass surgery, laparoscopic gastric band, laparoscopic sleeve gastrectomy leak, strangulated internal hernia

## Abstract

Internal hernia is a well-recognized complication after laparoscopic Roux-En-Y gastric bypass. Recently, it has been recognized after laparoscopic one-anastomosis gastric bypass. Alteration of bowel anatomy was put as the cause of internal hernia after these procedures.

Laparoscopic sleeve gastrectomy (SG) is one of the most commonly performed bariatric procedures worldwide; it was hypothesized that internal hernia could not occur after sleeve gastrectomy. We report in a video a case of internal hernia that occurred post laparoscopic sleeve gastrectomy and its concomitant management.

Data on the case of post-SG internal hernia were collected retrospectively and reported in a video with its intra-operative findings and concomitant management.

The patient is a 35-year-old male. He underwent laparoscopic sleeve gastrectomy, which was complicated by a leak. It was managed conservatively with optimal clinical response. He presented seven years after his surgery to the emergency department with a history of multiple episodes of severe left upper abdominal pain. a CT scan was performed, showing suspicion of an internal hernia. The patient was taken for emergency laparoscopic exploration.

Intra-operatively, there was a band of adhesion from a previous leak site connecting a loop of proximal jejunum to the anterior abdominal wall, forming a 5 cm defect. Through it, a loop of bowel was found herniating with partial twisting of its mesentery and engorgement of its vessels. In addition, there were multiple adhesions between bowel loops. The herniated bowel loop was reduced with no evidence of ischemia. The adhesive band was resected using a laparoscopic linear stapler. The bowel was fully inspected from the ileocecal valve up to the duodenojejunal (DJ) flexure, and a full adhesiolysis was performed.

The patient recovered well. He was discharged on day one postoperatively. He was followed up with a complete resolution of his symptoms and no complications.

We conclude that an internal hernia could occur as a long-term complication of sleeve gastrectomy leaks. A high index of suspicion should be applied when dealing with vague abdominal pain post-bariatric surgery. The laparoscopic approach was safe and feasible.

## Introduction

Bariatric surgery has had a rapid peak in development over the past 30 years for treating morbid obesity and its associated diseases [1]. With the high volume of bariatric surgery being performed, rare complications started to appear [2-9].

One of the well-reported complications is internal hernia post bariatric surgery, which occurred mainly post laparoscopic single-anastomosis gastric bypass (LOAGB) and laparoscopic Roux-En-Y gastric bypass (LRYGB) due to alteration of normal bowel and mesenteric anatomy [10-13]. Additionally, it was reported after laparoscopic adjustable gastric band (LAGB) [14].

Most of the cases of internal hernia post-bariatric surgery present with vague recurrent abdominal pain, which may lead to bowel gangrene if not diagnosed and treated early [15]. It was hypothesized that an internal hernia could not occur following laparoscopic sleeve gastrectomy (LSG).

This case was previously presented as a meeting abstract at the 2023 IFSO Annual Scientific Meeting on 30 August-1 September.

## Case presentation

Methods

We report a case of internal hernia occurring post laparoscopic sleeve gastrectomy and its concomitant management. The data of the case was retrospectively collected and reported in a video.

The case

A 35-year-old male who suffered from severe obesity with an initial BMI of 49.2 kg/m2, after a careful review of past medical history, showed no history of inflammatory bowel disease with low risk of fistula formation. He underwent laparoscopic sleeve gastrectomy. It was complicated by a leak for which it was treated conservatively by endoscopic stenting, image-guided drainage, and nutritional support. It had an optimal clinical response. The patient recovered well, and his BMI reached down to 26 kg/m2 two years after surgery.

The patient presented seven years following his surgery to our emergency department with vague abdominal pain. The pain was severe, colicky, and mainly in the left hypochondrial area. It was associated with nausea and occasional vomiting. There was no history of fever, constipation, or hematochezia. On physical examination, there was an abdominal tenderness over the left hypochondrial region; however, there were no signs of peritonitis. His vital signs were normal. Blood investigations showed normal leukocyte count and no rise in inflammatory markers.

A contrasted computerized tomography of the abdomen was done, and its results showed a mild mesenteric misty appearance, with the presence of a swirling sign that was highly suggestive of internal herniation.

Result

The patient was admitted and was taken for emergency laparoscopic exploration.

Under general anesthesia, the procedure was performed in a supine position. Pneumoperitoneum was achieved using optical entry via Palmer's point in the left upper quadrant, with gas insufflation. A total of four ports were inserted. Exploration of the abdominal cavity revealed an out-pouching structure arising from the anti-mesenteric border of the small bowel and attached to the anterior abdominal wall, forming a 5 cm defect. It was located 20 cm from the duodenojejunal (DJ) flexure. A loop of small bowel was found herniating through that defect with a partial twist of its mesentery and engorgement of its vessels. Careful inspection of the structure raised a high possibility of fistula formation. The structure was released from the anterior abdominal wall. Adhesions with amalgamation were found around that structure, which was dissected and released using sharp and blunt dissection.

Full bowel exploration was performed starting from the Ileocecal valve and heading towards the DJ flexure; unexplained adhesions were found between the terminal ilium, sigmoid colon, and proximal jejunal loop. A full adhesolysis was performed.

Finally, the fistula tract was completely resected using a laparoscopic liner stapler without jeopardizing the bowel lumen. A final full run of the bowel showed no bowel injury, internal hernia, or untreated adhesion. Ports sites were inspected for bleeding, gas was deflated, and no drain was inserted.

Post-operatively, the patient recovered well and was discharged on the first postoperative day. The patient was seen in the postoperative clinic after two weeks and six months with a complete resolution of his symptoms.

The final histopathology revealed a mature epithelial lining with no evidence of malignancy or inflammatory bowel disease 

Video 1 shows a summary of the case in video form.

**Video 1:** Video case report of an internal hernia post-laparoscopic sleeve gastrectomy

## Discussion

Internal hernia (IH) is a protrusion of the intestinal loop through a peritoneal or a mesenteric defect into another compartment of the abdominal cavity [16]. IH is well-recognized as a complication following RYGB. During laparoscopic RYGB, multiple mesenteric defects are created as a result of alteration of the gastrointestinal anatomy. These defects are a potential area for bowel herniation. IH may lead to bowel obstruction, strangulation, and eventually gangrene. Due to its vague clinical presentation and difficulty in detecting, IH carries a high morbidity and mortality rate [16]. Urgent surgery remains the definitive method of management. Routine closure of mesenteric defects has been described to decrease the incidence of IH [17].

During laparoscopic sleeve gastrectomy (LSG), the stomach size is decreased by vertically resecting the stomach over an oral gastric calibration tube without altering the normal anatomy of the bowel. Thus, it was hypothesized that IH could not occur after LSG. Nevertheless, stapler-line leaks are a well-recognized complication of LSG, with an incidence range between 0.5-3% [2]. Leaks cause intra-abdominal inflammation with the formation of adhesive bands and fistula tract formation that may play a role of a potential space for IH; however, no cases have been reported in the literature before to the best of our knowledge[12].

In our case, the patient developed IH with a bowel loop herniating through a defect caused by an inflammatory adhesive band, with no other potential mesenteric or peritoneal defect. To our best knowledge, this is the first reported case of internal hernia post laparoscopic sleeve gastrectomy, which had been complicated by a leak and treated conservatively in a morbidly obese patient. We have demonstrated that laparoscopic management of internal hernia is effective and safe.

## Conclusions

Although extremely rare, IH could occur after LSG with a history of staple-line leaks. A high index of suspicion of internal hernia should be raised in any vague abdominal pain following any bariatric surgery. A low threshold for urgent laparoscopic exploration in similar cases should be warranted. 
